# What family doctors know about congenital CMV: a regional survey in Iran

**DOI:** 10.1186/s13052-018-0470-4

**Published:** 2018-03-01

**Authors:** Ahmad Hosseinzadeh Adli, Chiman Karami, Sanaz Baghban Rahimi, Azam Mirarab, Alijan Tabarraei

**Affiliations:** 1 Department of Virology, Tehran University of Medical Sciences, Tehran, Iran; 2 Department of Virology, Ahvaz Joundishapoor University of Medical Sciences, Ahvaz, Iran; 3 Department of Microbiology, Golestan University of Medical Sciences, Gorgan, Iran; 4 Infectious diseases research center, Golestan University of Medical Sciences, Gorgan, Iran

**Keywords:** Congenital cytomegalovirus, cCMV, Awareness, Doctors, Iran

## Abstract

**Background:**

Since there is no effective treatment or vaccine against the congenital cytomegalovirus (cCMV) infection, knowledge and awareness of medical doctor’s (MDs) especially family doctors are essential for preventive strategies and it also seems to be usually ignored by healthcare providers. Aim of this study was to investigate awareness of MDs about cCMV infection in Iran.

**Methods:**

A single page questionnaire was randomly distributed among 450 MDs including general practitioners, pediatricians, gynecologists, internal and other medical specialists concerning of their knowledge in clinical presentation, diagnosis, prevention, prognosis, epidemiology, transmission, and management of cCMV infection. All statistical analyses were performed using SPSS version 16.

**Results:**

More than half of questionnaire recipients refused to take part in any of the questionnaire items. The most of the respondents were agreed for newborn CMV screening tests and mandatory CMV test for women trying to get pregnant, which, are not routinely tested. The knowledge of general practitioners about cCMV was less than usual. The field of expertise had a profound effect in this survey, but age and gender did not.

**Conclusions:**

Our results indicated that the knowledge of cCMV infection, especially among family doctors contains several gaps. Urgent action is required to improve family doctor’s knowledge of CMV infection. Surveys to evaluate CMV awareness among MDs, healthcare professionals and women of childbearing age are proposed.

**Electronic supplementary material:**

The online version of this article (10.1186/s13052-018-0470-4) contains supplementary material, which is available to authorized users.

## Background

Human cytomegalovirus (CMV) is an widespread virus and establishes lifelong latency following primary infection. Congenital CMV is one of the serious public health problems and the cause of the most common non-hereditary childhood hearing loss, but largely remains unrecognized in infants due to asymptomatic or nonspecific symptoms. Congenital CMV infection also is one of the common causes of neurodevelopmental delay, hearing loss and vision loss in developing countries. There are no symptoms at birth in about 90% of infected infants in an early period of life but about 10% of infants with cCMV infection will develop serious disorders, such as hearing loss, mental retardation, jaundice, seizures and microcephaly which have many side effects for lifetime. cCMV accounts for 20% to 25% of cases of sensorineural hearing loss (SNHL) and 30 to 35% will suffer of central nervous system (CNS) sequelae in developed countries. Unfortunately, for many parts of the world the maternal and birth CMV prevalence data are lacking, but certainly, the prevalence is higher in developing countries [1–7].

Since there is no effective vaccine against the cCMV infection yet, simple hygienic precautions remain the only successful strategy to prevent of cCMV infection. The rate of vertical transmission of CMV infection from mother to fetus is dependent on the time of mother’s infection [8–10]. Previous studies suggest that there is a limited awareness or knowledge about cCMV and prevention strategies among women who have educated by Family Practice Physicians and also it has been shown that delivery of prevention counseling to the pregnant women by healthcare providers were highly effective at raising awareness among pregnant women [11, 12]. Therefore, a successful preventive strategy depends on awareness of congenital CMV among MDs. There are insufficient data about the awareness and knowledge of cCMV infection among medical doctors and health care workers in developing countries. In order to investigate the clinician’s knowledge and awareness of cCMV, the responsiveness survey was carried out concerning clinical presentation, diagnosis, prevention, prognosis, epidemiology, transmission, and managemen of cCMV in MDs for the first time in Iran.

## Methods

This cross-sectional study was conducted in the North of Iran. A single page questionnaire on cCMV infection was randomly distributed among 450 family medical doctors to determine knowledge of cCMV. The questionnaire was anonymous and consisted of four parts, including demographic data, awareness, knowledge, and attitude about cCMV infection.

Demographic data regarding age, sex, educational level, professional field and work place were also asked. Finally, all questionnaires were collected and most were considered complete or almost complete when all important items were accepted to calculate. Incomplete and inconsistent responses to the questionnaire were excluded from the survey. In order to measure or determine the validity and reliability of the questionnaire using a pilot test, judgments of seven professors and expert in the scientific field of virology, clinical epidemiology and medical statistics were reviewed and analyzed. The questionnaire summary is given in Tables 1 and 2 as well as in Additional file 1.

**Table 1:** Summery of questionnaire and responses in three different groups

Knowledge concerning	General practitioners	Related medical specialties	Unrelated medical specialties	Total	*P* value
Transmission route True	86% (121)	94% (17)	90% (36)	87.88% (174)	0.556
False	14% (19)	6% (1)	10% (4)	12.12% (24)	
Symptoms in immune Competent adults	52. % (73) 48% (67)	83% (15) 17% (3)	73% (29) 28% (11)	59.09% (117) 40.91% (81)	0.006
Signs and symptoms in the	59% (82)	94% (17)	53% (21)	60.61% (120)	0.007
Neonatal period	41% (58)	6% (1)	48% (19)	39.39% (78)	
Long-term effects	59. % (83)	94% (17)	68% (27)	64.14% (127)	0.012
Sampling location	41% (57)	6% (1)	33% (13)	35.86% (71)	
	69% (97)	83% (15)	73% (29)	71.21% (141)	0.455
Confirmation test	31% (43)	17% (3)	28% (11)	28.79% (57)	
	25% (35)	22% (4)	45% (18)	28.79% (57)	0.039
Prevalence of symptomatic	75% (105)	78% (14)	55% (22)	71.21% (141)	
	34% (47)	72% (13)	50% (20)	40.40% (80)	0.003
Infection in infants	66% (93)	28% (5)	50% (20)	59.60% (118)	
Proper sampling time	60% (84)	89% (16)	68% (27)	64.14% (127)	0.049
	40% (56)	11% (2)	33% (13)	35.86% (71)

**Table 2:** Attitude questions and responses

Questions	Mandatory CMV test	Prenatal diagnostic test	Preventive interventions	Following infected infants	Newborn screening tests	Apparent disease in next child	Following virus shedding
Answers							
No idea	10.6% (21)	56.6% (111)	26.3% (52)	6.1% (12)	17.7% (35)	30.3% (60)	32.3% (64)
Certainly Disagree	5.6% (11)	9.09% (18)	4% (8)	2% (4)	5.6% (11)	31.3% (62)	10.1% (20)
Disagree	11.6% (23)	10.61% (21)	11.6% (23)	7.1% (14)	13.1% (26)	16.7% (33)	14.6% (29)
Agree	37.9% (75)	19.7% (39)	43.4% (86)	46% (91)	39.9% (79)	16.2% (32)	33.3% (66)
Certainly Agree	34.3% (68)	4.5% (9)	14.6% (29)	38.9% (77)	23.7% (47)	5.6% (11)	9.6% (19)

All statistical analyses were performed using SPSS version 16 (SPSSInc., Chicago, IL, USA), comparisons between different groups of respondents were made using SPSS Chi-Square test with the significance level set at (*P* value < 0.05).

## Results

The questionnaire was completed by 198 respondents. Among them, 70.7% (140) were general practitioner (family doctors) and 29.3% (58) were medical specialists. In medical specialists group, 9.1% [13] were responsible in the care of mothers and children and 20.2% (40) were as other specialist. The mean age of responders was 33.77 years. Of them, 34.7% were female and 62.6% were male, 82.9% of respondents were working in hospitals, 12.6% in administration offices, and 4.5% in health centers.

All related medical specialists, except one (6%), correctly identified modes of CMV transmission (True answers: blood contact and sexual intercourse, breastfeeding, kissing and changing diapers), (false answers: air conduction, direct skin, all items) and in the general practitioners and other medical specialists groups 86% and 90% gave true answer, respectively. No statistically significant differences were found between the groups (*p* value = 0.556).

In general practitioners group, 52% gave true answers about the symptoms in immune competent adults (True answers: without symptoms, elevated liver enzymes), (False answers: cardiac problems, visual problems, all items), and 83% and 73% of related and unrelated medical specialists knew the correct answer, respectively (*p* value = 0.006).

Research findings showed that growth restriction, microcephaly, and seizures were known as a neonatal symptom of cCMV by 94% of the related medical specialists, while only 53% of the unrelated medical specialists and 59% of the general practitioners knew the correct answer. About 39.3% of participants considered cardiac and renal diseases as cCMV infection wrongly. There were statistically significant differences between groups (*p* value = 0.007).

According to the findings, 94% of related medical specialists indicated correctly long-term effects of CMV congenital infection (True answers: hearing loss and visual problems, seizures, cognitive delay and dyspraxia), (false answers: risk of malignancy, no long-term effect, do not know) and 68 and 59% of unrelated medical specialists and general practitioners identified correct answer, respectively (*p* value = 0.003).

In general practitioners group, 69% gave true answers about proper specimen (True answers: saliva, urine, blood or amniotic fluid), (false answers: CSF, all items) and 83 and 73% of related and unrelated medical specialists knew the correct answer, respectively (*p* value = 0.455).

Nucleic acid testing (NAT) is the most sensitive method for the detection and confirmation of the cCMV infection and only 25, 22 and 45% of general practitioners, related and unrelated medical specialists gave true answers, respectively (*p* value = 0.039).

The prevalence of symptomatic cCMV infection is less than 20% in infected infants. According to the findings, 72% of related medical specialists, 50 and 72% of unrelated medical specialists and general practitioners identified correct answer, respectively. There were significant differences between groups (*p* value = 0.003).

The gold time for the quick and reliable diagnosis of cCMV infection in newborns is the detection of the virus within the first 2–3 weeks of life. In this study, 89% of related medical specialists, 68 and 60% of unrelated medical specialists and general practitioners identified correct answer, respectively (*p* value = 0.049).

However, gender issue and age did not have necessarily to be related to knowledge but the fields of expertise of participants did. As expected, most of medical specialists, especially, doctors involved in the care of mothers and children could identify answers correctly and awareness increased with higher level of education (*P* value< 0.05).

In attitude questions (Table 2 and Additional file 1), more than half of the respondents (63.6%) were strongly or somewhat agreed for newborn screening tests to detect genetic and metabolic disorders and hearing loss and also 72.2% of respondents were strongly or somewhat agreed to development mandatory CMV test for women trying to get pregnant. Only 4.5% of respondents were certainly agreed and 19.7% were somewhat agreed, that there is a prenatal diagnostic test for prognosis.

Most doctors (84.9%) believed that infants with established cCMV infection at birth should be at long-term follow-up to improve their physiological deficiencies. 14.6% of respondents were strongly agreed and 43.4% were agreed, that there is effective preventive interventions (vaccine and efficient therapeutic treatments) for CMV infection. Only 5.6% of respondents were certainly agreed that congenital CMV infection is hereditary diseases.

As in usual CMV is shedding in urine or saliva of congenitally infected infants in very high quantities the present study showed 33.3% of doctors were agreed and 6.6% were certainly agreed to measure virus titer at regular intervals in infected infants.

The Table 2 explains some of the attitudes of the respondents.

## Discussion

Congenital CMV is kept going by the lack of awareness, inaccessibility of an effective vaccine and efficient therapeutic treatments. However, the infection in pregnant women and subsequently, congenital infection can be markedly reduced with the use of knowledge about the nature of the virus and its ways of transmission. Poor training of health professionals is one of the main obstacles to reduce congenital CMV infection. The result of this study showed that awareness of congenital CMV infection among doctors was not in high quality at least on some aspects, and requires improvement.

Report in France showed that 46 and 23% of medical doctors did not recognize the transmission route and symptoms of CMV infection in newborns, respectively [14] while in Netherland only half of the respondent’s knew symptoms in newborns [15] .In comparison to above studies our survey demonstrated that doctors were more aware and possess optimal knowledge however, knowledge of CMV infection among doctors in the North of Iran contained some gaps, including attitudes towards preventive methods, prevalence, and paraclinical information.

Our results showed nearly half of the medical doctors knew wrongly, about application of effective prophylactic drug treatments during pregnancy. CMV hyperimmune globulin in pregnant women may prevent some congenital infection, but safety and efficacy of this treatment and antiviral drugs have not been established and are not routinely used [16, 17] .Until now, no effective intervention is designed to cope with the cCMV infection. Therefore, medical doctors must routinely counsel patients about CMV preventive methods and behavioral interventions before pregnancy. In developing countries, many of women in reproductive age are CMV seropositive [18] .A common source of CMV transmission to a pregnant woman is via close contact with infected body fluids (urine, saliva), frequently from young children, or from household contacts. During viral reactivation/reinfection or primary infection in pregnant women CMC can be transmitted to the fetus. A method of preventing cCMV is through hygienic practices, therefore, developing educational projects to promote effective hygiene is very important [13] .Health care professionals can play a critical role in disseminating information about preventive methods of c CMV infection to the women of childbearing age. Giving hygiene information to high-risk women in childbearing age may be highly effective in preventing CMV infection [19] .In the French survey, about 20% of the respondents (44% in medical doctors) knew there is available treatment during pregnancy while it was 34.6% in Netherlands (55.3% in pediatrics and 27.8% in obstetrics and gynecologists) as it was 58% in our survey [14, 15] .It seems designing strategies for changing the doctor’s attitudes towards preventive methods is necessary.

Congenital CMV infection is more common than many metabolic and endocrine disorders in infants. Less than 20% of cCMV infections in children develop disabilities. The universal newborn screening (blood, urine and saliva specimens) would be improve language and developmental outcomes through early intervention, but costs and harms must be considered [20, 21] .In this study, more than half of the doctors were somewhat or certainly agree to newborns screening program. Some of the European countries routinely screen the majority of pregnant women serologically for CMV without the recommendations or guidelines of any governmental agency [22] .Screening is not diagnostic; rather, it identifies individuals who should be referred for confirmatory diagnostic test. A positive screening result requires a subsequent diagnostic evaluation. Accurate method for diagnosis of CMV infection, such as a low avidity anti-CMV IgG antibodies and nucleic acid test are very expensive in our country and some women who decided to get pregnant might oppose mandatory CMV test and considered the potential costs of screening. However, Cahill AG and colleagues showed the universal screening for primary maternal cytomegalovirus infection is cost-effective [23] then, healthcare professionals must provide counsel of women in reproductive age about CMV screening. It is necessary to consider whether voluntary tests with informed decision would be more appropriate than mandatory test.

In developing countries, follow-up of infected infants is not performed because of financial costs. However, more than half of the doctors have a positive attitude towards establishment of these kinds of programs as soon as possible.

The most significant limitation of the present study was a very low response rate to the questionnaires. Since respondents may do not care enough or respondents who felt unsure about their knowledge of CMV infection could refuse to respond to the questionnaire, it might imply that the true knowledge concerning congenital CMV infection is even poorer than reported. Large-scale surveys could be a good solutions to solve this problem.

The knowledge of cCMV infection, especially among general practitioners in North of Iran has some gaps. In this study, medical doctors seem to have low level of knowledge on the cCMV infection, symptoms in healthy adults and know less about preventive interventions as well as less information about the paraclinical aspects. This leads to poor management of children with cCMV infection.

## Conclusions

In developing countries conducting surveys to evaluate CMV awareness among medical doctors, healthcare professionals, and women of childbearing age are necessary. Successful programs and methods to prevent, diagnose or reduce the cCMV infection will require global awareness among clinicians, pregnant women, medical and non-medical staff for early detection and intervention for preventive strategies and reducing clinical outcomes.

## Additional file

Additional file 1: Questionnaire ‘What family doctors know about congenital CMV: a regional survey in Iran’. (DOC 24 kb)

## Abbreviation

CMV: Cytomegalovirus
