# Supplementary material for: What family doctors know about congenital CMV: a regional survey in Iran
Source: Ital J Pediatr. 2018 Mar 1;44:31. doi: 10.1186/s13052-018-0470-4 (PMC5831851; doi:10.1186/s13052-018-0470-4)
Supplement: Supplementary file 1 — Questionnaire ‘What family doctors know about congenital CMV: a regional survey in Iran’. (DOC 24 kb) [file 13052_2018_470_MOESM1_ESM.doc]

**Questionnaire " What family doctors know about congenital CMV: a regional survey in Iran"**

**Section A: Knowledge questions**

1- What is the transmission route of CMV infection?

2- What is the most frequent presentation of CMV infection in immune competent adults?

3- What symptoms can be seen in newborns with congenital CMV infection?

4- What long-term effects can present in children with congenital CMV infection?

5- What are the Ideal specimens for congenital CMV tests?

6- What is the standard laboratory test for confirmation of congenital CMV infection?

7- What is the prevalence rate of symptomatic Infection in infected infants?

8- When is the proper sampling time in infected infants?

**Section B: Attitude questions**

1- What is your opinion about the establishment of mandatory CMV test for women of childbearing age?

2- In your opinion, is there a prenatal diagnostic test for prognosis?

3- In your opinion, are there effective therapeutic interventions (vaccine and efficient therapeutic treatments) for prevention?

4- In your opinion, follow-up of infected infants is necessary?

5- What is your opinion about newborn screening tests?

6- In your opinion, congenital CMV infection is hereditary diseases?

7- What is your opinion about Follow-up virus shedding in infected infants?
